# Prolonged Screen Viewing Times and Sociodemographic Factors among Pregnant Women: A Cross-Sectional Survey in China

**DOI:** 10.3390/ijerph15030403

**Published:** 2018-02-27

**Authors:** Xianglong Xu, Dengyuan Liu, Yunshuang Rao, Huan Zeng, Fan Zhang, Lu Wang, Yaojie Xie, Manoj Sharma, Yong Zhao

**Affiliations:** 1 School of Public Health and Management, Chongqing Medical University, Chongqing 400016, China; xianglong1989@126.com (X.X.); dengyuanliu@foxmail.com (D.L.); rys0606@163.com (Y.R.); zenghuan586@aliyun.com (H.Z.); ava11@126.com (F.Z.); wl532916490@163.com (L.W.); 2 Research Center for Medicine and Social Development, Chongqing Medical University, Chongqing 400016, China; 3 Collaborative Innovation Center of Social Risks Governance in Health, Chongqing Medical University, Chongqing 400016, China; 4 School of Nursing, The Hong Kong Polytechnic University, Hong Kong 999077, China; yaojie.xie@gmail.com; 5 Department of Behavioral and Environmental Health, Jackson State University, Jackson, MS 39213, USA; manoj.sharma@jsums.edu.cn

**Keywords:** screen time, TV viewing, computer viewing, mobile phone viewing, pregnant women, China

## Abstract

*Objectives:* This study aimed to assess the prevalence of prolonged television, computer, and mobile phone viewing times and examined related sociodemographic factors among Chinese pregnant women. *Methods:* In this study, a cross-sectional survey was implemented among 2400 Chinese pregnant women in 16 hospitals of 5 provinces from June to August in 2015, and the response rate of 97.76%. We excluded women with serious complications and cognitive disorders. The women were asked about their television, computer, and mobile phone viewing during pregnancy. Prolonged television watching or computer viewing was defined as spending more than two hours on television or computer viewing per day. Prolonged mobile phone viewing was watching more than one hour on mobile phone per day. *Results:* Among 2345 pregnant women, about 25.1% reported prolonged television viewing, 20.6% reported prolonged computer viewing, and 62.6% reported prolonged mobile phone viewing. Pregnant women with long mobile phone viewing times were likely have long TV (Estimate = 0.080, Standard Error (*SE*) = 0.016, *p* < 0.001) and computer viewing times (Estimate = 0.053, *SE* = 0.022, *p* = 0.015). Pregnant women with long TV (Estimate = 0.134, *SE* = 0.027, *p* < 0.001) and long computer viewing times (Estimate = 0.049, *SE* = 0.020, *p* = 0.015) were likely have long mobile phone viewing times. Pregnant women with long TV viewing times were less likely to have long computer viewing times (Estimate = −0.032, *SE* = 0.015, *p* = 0.035), and pregnant women with long computer viewing times were less likely have long TV viewing times (Estimate = −0.059, *SE* = 0.028, *p* = 0.035). Pregnant women in their second pregnancy had lower prolonged computer viewing times than those in their first pregnancy (Odds Ratio *(OR)* 0.56, 95% Confidence Interval *(CI)* 0.42–0.74). Pregnant women in their second pregnancy were more likely have longer prolonged mobile phone viewing times than those in their first pregnancy (*OR* 1.25, 95% *CI* 1.01–1.55). *Conclusions:* The high prevalence rate of prolonged TV, computer, and mobile phone viewing times was common for pregnant women in their first and second pregnancy. This study preliminarily explored the relationship between sociodemographic factors and prolonged screen time to provide some indication for future interventions related to decreasing screen-viewing times during pregnancy in China.

## 1. Introduction

Prolonged screen viewing times are common globally. Prolonged screen viewing times have been documented as prevalent sedentary behaviors associated with an increased risk of adverse pregnancy outcomes and chronic diseases. The association is found between prolonged mobile phone viewing and low birth weight, high infant emergency transfer rate [1], and shorter pregnancy duration [2]. Prolonged screen viewing times are also adversely associated with physical well-being, mental well-being, and vitality [3]. Obesity [4,5], developing diabetes [6], hypertension [7], and several musculoskeletal health complaints [8] are also associated with long screen viewing times. In addition, prolonged mobile phone usage is associated with delayed bedtime, short sleeping time [9,10], brain tumors, and parotid gland tumor [11]. Television viewing times have an inverse association with adjusted telomere length among Southwest Chinese adults [12].

According to the 2002 Chinese residents of nutrition and health survey, about 92.1% of Chinese residents watch TV, with an average time of 2.1 h every day [13], and 60.6% of these Chinese residents watch TV for over 2 h daily [14]. Approximately 59% of American adults aged 20 years and older watch over 2 h of TV daily [15]. Computer usage has become one of the most prevalent factors affecting screen viewing times in China [16]. The average computer exposure time of a Chinese female is 2.9 h/day during 2011–2012 [14]. Mobile phone subscribers will increase from 4.8 billion in 2016 to 5.7 billion in 2020 globally [17]. In 2016, more than half of Chinese people used mobile phone more than 2 h per day [18].

Sociodemographic factors, such as age [14,19], gender [10,14,20,21], income level and labor intensity [22], educational level [19,23], residence [24], transportation options [25], business setting [26,27], and consumption level and frequency of use of the entertainment functions [10] are associated with prolonged screen viewing times. Previous studies have explored some sociodemographic factors related to prolonged screen viewing times, primarily among adults and children. Moreover, with the issuance of the Chinese new policy “two-child policy” in 2015, the number of pregnant women will decrease. Thus, the present study incorporates related factors, including parity and trimester, apart from sociodemographic factors.

To our knowledge, the prevalence of prolonged screen viewing times among pregnant women is unclear, and few studies have focused on the sociodemographic factors of prolonged screen viewing times among pregnant women, especially in China. This study provides an improved implication and understanding of the policies in Healthy China 2030 and National Nutrition Program by optimizing a healthy lifestyle among pregnant women. This study aims to assess prolonged TV viewing times, computer usage times, mobile phone viewing times, and their influencing factors (socioeconomic characteristics factors, trimester, and parity) among Chinese pregnant women to solve the following three objectives:

- To determine the prevalence rate of prolonged TV viewing, computer usage, and mobile phone viewing times among pregnant women in China;
- To indicate the association among prolonged TV viewing, computer usage, and mobile phone viewings time;
- To identify the factors affecting prolonged TV viewing, computer usage, and mobile phone viewing times among Chinese pregnant women.

## 2. Materials and Methods 

### 2.1. Study Design and Participants

The design and methods, sampling framework, survey administration, and pilot study, as well as the population and sample questionnaire development process, of the current study have been reported previously [28,29]. In brief, a total of 2400 pregnant women were surveyed by trained medical students, with a response rate of 97.76%. Women with serious complications and cognitive disorders were excluded. All pregnant women visiting 16 hospitals in Chongqing, Chengdu, Zunyi, Liaocheng, and Tianjin were invited to participate in the survey from June to August in 2015. Chongqing, Chengdu, and Zunyi are in south China, whereas Liaocheng and Tianjin are in north China. The study protocol was ratified by the Ethics Committee of Chongqing Medical University (record number 2015008). All subjects provided their informed consent for inclusion before their participation.

### 2.2. Questionnaire

#### 2.2.1. Sociodemographic Variables

Sociodemographic data included residence (Urban/Rural) and per capita monthly income of the family (low income (<¥4500)/medium income families (¥4500–¥9000)/high income families (>¥9000)), (1 USD = ¥6.79 in June 2017)). Other categories included age (18–25 years/26–35 years/36–45 years), occupation (rural migrant workers/urban and rural unemployed, unemployed/industrial workers of non-agricultural registered permanent residence/individual business/business services staff/civil servants/senior and middle-level managers in large and medium enterprises/private entrepreneur/professionals/clerks/students/others), hospital capacity/quality rank (high, medium, and low), nationality (Han nationality/minority), without sibling (yes/no), marital status (unmarried/married/remarried/divorced/widowed). Pregnancy was divided into three trimesters (first, second, and third trimester). Education level was categorized as low (junior, middle school, or below), medium (senior high school, vocational or technical secondary school), or high (university). Based on the Chinese hospital ranking system, the hospital capacity/quality rank was recorded as high, medium, or low [30].

In the multivariable analysis on watching over two hours of TV daily, computer usage times of more than two hours per day, and mobile phone viewing times of more than one hour per day, occupational category was categorized as manual (rural migrant workers/industrial workers of non-agricultural registered permanent residence/business services staff), non-manual (individual business/civil servants/senior manager and middle-level manager in large and medium enterprise/private entrepreneur/professionals/clerk/students), or unemployed.

#### 2.2.2. Outcome Variables

Participants were asked about their total TV viewing, computer usage (including computer time at work), and mobile phone viewing times daily during pregnancy per day. Compared with adults without health conditions, a higher percentage of adults with health conditions watched over two hours of TV and computer per day [31]. Exposure to mobile phone usage for more than 1 h daily and parotid tumor was observed to be associated [32]. Prolonged watching times were defined as when a woman watched over two hours on TV or computer and watched over one hour on mobile phone per day. Therefore, participants were divided into three categories: TV viewing times (more than two hours per day and less than two hours per day), computer usage times (more than two hours per day and less than two hours per day), and mobile phone viewing times (more than one hour per day and less than one hour per day).

### 2.3. Statistical Analyses

Participants’ characteristics were summarized using either the means and standard deviations or the frequencies and percentages, which were presented using descriptive analysis (means, standard deviations, and percentages). Generalized linear model was utilized to probe factors that affected TV, computer, and mobile phone viewing times. Factors that extended TV, computer, and mobile phone viewing times were considered in the generalized linear model among pregnant women: TV viewing times, mobile phone usage times, computer usage times, parity, hospital level, occupational category, nationality, being a single-child, husband being a single-child, marital status, educational level, residence, age, trimester, and per capita monthly income of the family.

We included mobile phone usage times, hospital level, occupational category, without siblings, per capita monthly income of the family, and ”prolonged TV viewing times” as dependent variables in the multivariate logistic regression model with backward elimination. Multivariate model was statistically significant in the model coefficient test (*p* < 0.05) and reached a good fit in Hosmer and Lemeshow test (*p* = 0.78). We included mobile phone viewing times, age, hospital level, occupational category, without siblings, education level, residence, per capita monthly income of the family, and trimester of pregnancy with “prolonged computer usage times” as the dependent variable in the multivariate logistic regression model with backward elimination; multivariate model was statistically significant in the model coefficient test (*p* < 0.05) and reached a good fit in Hosmer and Lemeshow test (*p* = 0.25). We included TV viewing times, computer usage times, age, occupational category, marital status, education level, and trimesters of pregnancy with “prolonged mobile phone usage times” as dependent variables in the multivariate logistic regression model with backward elimination. Multivariate model was statistically significant in the model coefficient test (*p* < 0.05) and reached a good fit in Hosmer and Lemeshow test (*p* = 0.54). All statistics were performed using 2-sided test, and statistical significance was considered at *p* < 0.05. All data analyses were performed using statistical software (SAS version 9.1.3; SAS Institute, Cary, NC, USA).

## 3. Results

### 3.1. Characteristics of Pregnant Women 

Of the 2345 participants (28.12 ± 4.1 years), 1755 (74.8%) were in their first pregnancy, and 590 (25.2%) are in their second pregnancy. About 96% of pregnant women were of Han nationality. (Please see Table 1).

### 3.2. Screen Times of Pregnant Women

Daily TV viewing times of between two and four hours were reported by 12.5% of respondents. Among pregnant women, 9.4% watched TV for 4–6 h and 3.1% spent more than 6 h viewing TV daily.

Daily computer viewing times between two and four hours were 4.6%. Among pregnant women, 6.4% watched computer for 4–6 h and 9.6% spent more than 6 h on computer viewing per day.

Daily mobile phone usage times between one and two hours were 26.5%. Among pregnant women, 14.3% viewed mobile phones for 2–4 h, 13.8% viewed mobile phones for 4–6 h, and 7.9% spent more than 6 h on mobile viewing per day (see Table 2).

### 3.3. Prolonged TV Viewing, Computer Usage, and Mobile Phone Usage Times among Pregnant Women 

Prolonged TV viewing times were reported by 456 (26.0%) and 132 (22.4%) participants on their first and second pregnancies, respectively. A total of 466 (24.8%) and 122 (26.2%) participants form urban and rural areas, respectively, were reported to watch over two hours of TV per day (see Table 3).

Prolonged computer usage times were reported by 405 (23.1%) and 77 (13.1%) participants on their first and second pregnancies. A total of 55 (11.8%) and 427 (22.7%) participants from urban and rural areas, respectively, were reported to watch computer more than 2 h daily (see Table 3).

Prolonged mobile phone usage times were reported by 1081 (61.6%) and 386 (65.4%) participants on their first and second pregnancies. A total of 1172 (62.3%) and 295 (63.4%) participants from urban and rural areas, respectively, were reported to watch over one hour of mobile phone per day (see Table 3).

### 3.4. Generalized Linear Model for Long TV, Computer, Mobile and Total Viewing Time among Pregnant Women, China

Generalized linear model found that pregnant women in their second pregnancy (*p* = 0.0009) had high level of education (*p* = 0.008) and were less likely have long TV viewing times. Pregnant women whose family’s per capital monthly income was more than ¥9000 (*p* = 0.020) and those who were 26–35 years old (*p* = 0.007) and 36–45 years old (*p* = 0.026) tended to have long TV viewing times.

Generalized linear model indicated that pregnant women in their second pregnancy (*p* < 0.001) from level 2A hospitals (*p* = 0.005), of manual (*p* = 0.040) and unemployed (*p* < 0.001), were less likely to have long computer usage times. Pregnant women with high level of education (*p* = 0.0002) and those aged 26–35 years old (*p* = 0.010) tended to have long computer usage times.

Generalized linear model found that pregnant women of manual (*p* = 0.001), unemployed (*p* = 0.012), and with high level of education (*p* = 0.047) were less likely to have long mobile phone viewing times. Pregnant women in their second pregnancy (*p* = 0.041) tended to have long mobile phone viewing times.

Generalized linear model showed that pregnant women in their second pregnancy (*p* = 0.006), manual (*p* = 0.002), and unemployed (*p* = 0.0001) were less likely have long screen times. Pregnant women of primary marriage (*p* = 0.008) and remarried (*p* = 0.018) tended to have long screen viewing times (see Table 4).

### 3.5. Multivariate Logistic Regression Model for TV Viewing Times of More Than Two Hours Daily

Pregnant women with more than one hour of mobile phone usage time were more likely to watch over two hours of TV per day than pregnant women with less than one hour of mobile phone usage [*OR* = 1.52, 95% *CI*: 1.25−1.86]. Unemployed pregnant women [*OR* = 1.65, 95% *CI*: 1.30−2.10] and marked others [*OR* = 1.42, 95% *CI*: 1.064−1.89] tended to watch over two hours of TV daily. Pregnant women without siblings were less likely to have prolonged TV viewing times than those with siblings [*OR* = 0.79, 95% *CI*: 0.65−0.95]. Pregnant women whose family’s per capita income was more than ¥9000 were likely watch over two hours of TV per day [*OR* = 1.42, 95% *CI*: 1.10−1.86] (see Table 5).

### 3.6. Multivariate Logistic Model for Computer Usage Times of More Than Two Hours per Day

Pregnant women in their second pregnancy were less likely to have over two hours of computer viewing time per day than those in their first pregnancy [*OR* = 0.56, 95% *CI*: 0.42−0.74]. Pregnant women from Level 2A hospitals tended to have over two hours of computer viewing time per day [*OR* = 0.53, 95% *CI*: 0.31–0.89]. Unemployed pregnant women [*OR* = 0.51, 95% *CI*: 0.37−0.70] were less likely to have over two hours of computer viewing time daily. Pregnant women with completed high education tended to have computer viewing times of over two hours per day [*OR* = 2.72, 95% *CI*: 1.78−4.16]. Pregnant women without siblings were less likely to have prolonged computer viewing times than those with siblings [*OR* = 0.76, 95% *CI*: 0.61−0.94]. Pregnant women whose family’s per capita income was between ¥4500 and ¥9000 were less likely have over two hours of computer viewing time per day [*OR* = 0.69, 95% *CI*: 0.52−0.92] (see Table 5).

### 3.7. Multivariate Logistic Model for Mobile Phone Usage Times of More Than One Hour Daily 

Pregnant women with more than two hours of TV viewing time were more likely to have over one hour of mobile phone viewing time per day than those with less than two hours of TV viewing time [*OR* = 1.56, 95% *CI*: 1.27−1.91]. Pregnant women aged between 26 to 35 years tended to have over one hour of mobile phone viewing time per day [*OR* = 0.80, 95% *CI*: 0.65−0.98]. Pregnant women in their second pregnancy were more likely to have prolonged mobile phone viewing times than those in their first pregnancy (*OR* = 1.25, 95% *CI*: 1.01−1.55) (see Table 5).

## 4. Discussion

Prolonged television, computer, and mobile phone viewing times are common during pregnancy. Clarifying the factors affecting screen-viewing times during pregnancy is necessary to decrease it through educational behavior change interventions. A previous national study found that among Chinese women, prolonged television viewing time prevalence was high (69.3%) in 2002 [13] and 2013–2015 (62.63%) [12], and these prevalence rates are higher than those among pregnant women of the present study; however, the prevalence rate of the present study still has a high number for pregnant women and may increase the number of adverse pregnancy outcomes. On 29 October 2015, the Chinese government announced the “two-child policy”, implying that more Chinese women will be impregnated. The present study found that high-risk groups for prolonged mobile phone viewing were those in their second pregnancy, although they were less likely have prolonged computer viewing times. Further measures are necessary to achieve the target of Health China 2030, which promotes healthy lifestyles and eliminates health-related risk factors [33]. This study has implications in the implementation and enforcement of Health China 2030 that promotes healthy lifestyles and eliminates health-related risk factors. An American family-based intervention in women, infants, and children [34] may indicate that a family-based intervention of increasing outdoor playtime may be also feasible and efficient among pregnant women.

The present findings indicated a mutual correlation between prolonged TV and mobile phone viewing times. Pregnant women with prolonged mobile phone viewing times were more likely to have prolonged TV viewing times. A similar study also found that pregnant women who used both mobile phones and computers were more likely to have a short pregnancy duration [2]. This result may indicate that pregnant women with prolonged mobile usage have a more positive attitude towards electronic products including television compared with those without prolonged mobile phone usage times. This finding may be associated with the multiple and advanced functions of TV nowadays, whereby people can watch films, TV series, live online shows, and even play games [35]. Pregnant women can enjoy similar experiences and advanced features through mobile phone and TV, such as watching movies and popular soap operas to spend daily leisure time. As applications that can connect to and control TV are continuously being developed, pregnant women can use mobile phones when watching TV, which may also increase the likelihood of prolonged TV viewing times of pregnant women who enjoy using mobile phones. Thus, the effect of the screen viewing of various electronic products should be investigated in the future. Pregnant women with prolonged TV or mobile phone viewing times tended to suffer greater adverse health outcomes. Moreover, pregnant women whose single screen viewing times on one of the modalities were not prolonged may have a prolonged combined total viewing time.

A high-risk group with prolonged mobile phone viewing times is those women in their second pregnancy. Interestingly, they are also less likely to have prolonged computer viewing times. Decreased phone usage among pregnant women during their first pregnancy may be attributed to them being cautious and to their families who were likely to reduce mobile phone usage. Thus, the great potential need for surfing the Internet to seek pregnancy-related knowledge and other online information may increase possibility of computer viewing [36]. However, pregnant women in their second pregnancy with prior gestational experience may not be as cautious as those in their first pregnancy, including the reduced frequency of prenatal examination [37]. A previous study found a high rate of information seeking behaviors through the Internet given that 88.7% of pregnant women used the Internet to learn about health-related information, in which fetal development and pregnancy nutrition knowledge were the two most often mentioned topics [36]. Additionally, pregnant women in their first pregnancy were usually younger than those in their second pregnancy, and given that younger women viewed computers for over two hours more often in China [38], this may explain the finding from another perspective.

Pregnant women with siblings tended to have prolonged TV and computer viewing times. This scenario may indicate the positive influence of siblings on pregnant women in prolonged TV and computer viewing times, and a previous study has also found the influence of siblings on the insufficient sleep of pregnant women in China [39]. Future studies should focus on pregnant women with siblings and explore the influence of siblings on their family members regarding prolonged screen viewing times and other related health behaviors. Prolonged screen viewing times are definitely unhealthy, but we cannot deny their convenience and instantaneity, and a technology-based e-health intervention may be convenient and efficient at reducing the frequency of prolonged TV and computer viewing times [40], for example, daily health message and timing reminder software.

Furthermore, women with high monthly income and who were unemployed showed an increased prevalence of prolonged TV viewing times during pregnancy. Pregnant women with high income likely have prolonged television viewing times. Similar to a previous Singaporean study, pregnant women with high incomes are likely have high sitting times, including watching TV and low reduction in physical activity [41]. Those with high monthly income possibly have low economic pressure and less housework. Likewise, pregnant women with high monthly income have more money to hire nannies and are more likely to stay at home for safety, giving them sufficient time to watch TV. By contrast, pregnant women with low monthly income cater to their family or work for more money, which may explain this finding. The present study also found that unemployed pregnant women likely watched television for long periods of time. The possible reason is that those pregnant women need more time to use television for leisure time. With the rise of Chinese economic levels, the number of pregnant women with high monthly income will further increase. Health professionals should focus on these pregnant women, and measures should also be taken to intervene in the prolonged TV watching times.

Additionally, pregnant women with high educational level aged 26–35 years old show increased prevalence of prolonged computer viewing times. Pregnant women with complete higher education likely watch computers for over two hours per day, and a previous national survey in China found higher prevalence rate of computer usage among women from urban areas than those from rural areas [42]. Most pregnant women who are senior intellectuals may be mental workers, and possibly have higher acceptance and usage habits in general life and work for computer usage compared with pregnant women with low educational level. Additionally, those pregnant women may undertake significant business in their institutions and whether or not they needed the assistance of computers demands further attention. A previous Chinese study also found that pregnant women with high education level regarded health-related information from the Internet as believable and dependable [36]. This previous finding may indicate that pregnant women with higher educational level have high frequencies of information-seeking behavior through computers. The present finding may reveal a key situation that pregnant women of higher educational level tended to have high possibility of prolonged computer viewing times. Health-related workers and future studies must focus on those pregnant women, and other populations with higher educational level may also need investigation. Family-work unit-based interventions may be necessary for those pregnant women. Pregnant women with siblings were more likely to have prolonged TV viewing times and prolonged computer viewing times than those without siblings, which may indicate the influence of siblings on prolonged TV and computer viewing times. Reasons for the present finding are multifaceted and synthetic; thus, future studies should focus on this phenomenon. In any case, given the issue of “two-child policy”, families adhering to this policy will increasingly appear in China, and the present finding will be worse than before. Efficient preventive measures should be taken to reduce the prevalence among pregnant women that have siblings.

Pregnant women with basic educational level, low age, and primary marriage showed an increased prevalence of prolonged mobile phone viewing times. Pregnant women with basic educational level and who are younger presented more chances of prolonged mobile phone viewing times. Furthermore, previous research indicates that young women with low education tended to spend long time on each call [19], and a Japanese survey also showed that young pregnant women with low educational level likely use their mobile phones excessively [1]. Young pregnant women are possibly intrigued by mobile phones. Besides, pregnant women with basic educational level may have less amateur activities for their limited knowledge and opportunities compared with those of general educational level. Pregnant women with excessive mobile phone usage prefer to hide their phones in their pockets or other places for convenience [1]; thus, those pregnant women may be exposed to increased radiation. The present study also found that unmarried pregnant women were less likely to have prolonged mobile phone usage, which differs from a previous study in which single pregnant women tended to have excessive usage of mobile phones [1]. Thus, further studies should be based on ethnic, regional, and socioeconomic status to confirm the high risk population in China.

The present study also has certain notable limitations. First, the cross-sectional survey data prevented researchers from making direct causal inferences, exploring whether or not the unmeasured factors may explain the observed relationships, and determining the direction of causality. Second, the face-to-face survey administration design and self-reported screen-viewing times may have introduced some information and measurement bias. Third, our study is not precisely nationally representative. The sample comprised the population of pregnant women in five regions of China, namely, Chongqing, Chengdu, Zunyi, Liaocheng, and Tianjin. Chongqing, Chengdu, and Zunyi are in southern China, while Liaocheng and Tianjin are in northern China. No evident difference is observed between western, eastern, and central China relative to the proportion of pregnant women that watch TV [43].

## 5. Conclusions

Pregnant women have high a prevalence rate of prolonged TV, computer, and mobile phone viewing times, which is common for those in their first and second pregnancies. Findings provide a primary and exploratory understanding of the prolonged television, computer, and mobile phone viewing times among Chinese pregnant women. This study outlines some of the implications of decreasing screen-viewing times during pregnancy in China, and these findings may concentrate on the implementation and development of the “Healthy China 2030 policy”.

## Figures and Tables

**Table 1:** Characteristics of the study participants in Chongqing, China, 2015 (*n*, %).

Variable	Frequency	Percentage
Age		
18–25 years old	624	26.6
26–35 years old	1595	68.0
36–45 years old	126	5.4
Parity		
Pregnant women in first pregnancy	1755	74.8
Pregnant women in second pregnancy	590	25.2
Hospital level		
Level 3A hospital	1824	77.8
Level 2 A hospitals	311	13.2
Level 2B hospitals and below	210	9.0
Nationality		
Han nationality	2252	96.0
Non-Han nationality	93	4.0
Without siblings		
Yes	1046	44.6
No	1299	55.4
Husband without siblings		
Yes	1173	50.0
No	1172	50.0
Marital status		
Unmarried	49	2.1
Primary marriage	2205	94.0
Remarried	70	3.0
Divorced or Widowed	21	0.9
Education level		
Basic education	402	17.1
Secondary education	354	15.1
Higher education	1589	67.8
Residence		
Urban	1880	80.2
Rural	465	19.8
The per capital monthly income of the family		
<¥4500	611	26.0
¥4500 and ¥9000	989	42.2
>¥9000	745	31.8
Occupation		
Rural migrant workers	118	5.0
Urban and rural unemployed	553	23.6
Industrial workers of Non-agricultural registered permanent residence	50	2.1
Individual business	199	8.5
Business services staff	155	6.6
Civil servants	398	17.0
Senior managers and Middle-level managers in large and medium enterprises	96	4.1
Private entrepreneur	87	3.7
Professionals	244	10.4
Clerk	139	5.9
Students	15	0.7
Other	291	12.4
Occupational category		
Non-manual	1178	50.2
Manual	323	13.8
Unemployed	553	23.6
Others	291	12.4
Trimester of pregnancy		
First trimester	293	12.5
Second trimester	701	29.9
Third trimester	1351	57.6

**Table 2:** Screen times of pregnant women in Chongqing, China (*n*, %).

Total Screen Time (5.90 ± 3.70 h)	TV Viewing Times	Computer Viewing Times	Mobile Viewing Times
Mean ± SD	1.80 ± 1.68 h	1.65 ± 2.32 h	2.45 ± 2.18 h
≤1	1134 (48.4)	1572 (67.0)	878 (37.4)
(1, 2]	623 (26.6)	291 (12.4)	622 (26.5)
(2, 3)	14 (0.6)	5 (0.2)	14 (0.6)
[3, 4)	280 (11.9)	102 (4.4)	322 (13.7)
[4, 5)	137 (5.8)	83 (3.5)	203 (8.7)
[5, 6)	84 (3.6)	68 (2.9)	120 (5.1)
≥6	73 (3.1)	224 (9.6)	186 (7.9)

**Table 3:** TV viewing, computer using and mobile phone usage times among pregnant women, China, 2015 (*n*, %).

Variable	TV Viewing Times		Computer Usage Times		Mobile Phone Viewings	
	≤2 h/day	＞2 h/day	≤2 h/day	>2 h/day	≤1 h/day	>1 h/day
Age						
18–25 years old	481 (77.1)	143 (22.9)	528 (84.6)	96 (15.4)	217 (34.8)	407 (65.2)
26–35 years old	1187 (74.4)	408 (25.6)	1228 (77.0)	367 (23.0)	614 (38.5)	981 (61.5)
36–45 years old	89 (70.6)	37 (29.4)	107 (84.9)	19 (15.1)	47 (37.3)	79 (62.7)
Parity						
Pregnant women in first pregnancy	1299 (74.0)	456 (26.0)	1350 (76.9)	405 (23.1)	674 (38.4)	1081 (61.6)
Pregnant women in second pregnancy	458 (77.6)	132 (22.4)	513 (86.9)	77 (13.1)	204 (34.6)	386 (65.4)
Hospital level						
Level 3A hospital	1353 (74.2)	471 (25.8)	1418 (77.7)	406 (22.3)	700 (38.4)	1124 (61.6)
Level 2 A hospitals	248 (79.7)	63 (20.3)	280 (90.0)	31 (10.0)	103 (33.1)	208 (66.9)
Level 2B hospitals and below	156 (74.3)	54 (25.7)	165 (78.6)	45 (21.4)	75 (35.7)	135 (64.3)
Nationality						
Han nationality	1684 (74.8)	568 (25.2)	1783 (79.2)	469 (20.8)	845 (37.5)	1407 (62.5)
Non-Han nationality	73 (78.5)	20 (21.5)	80 (86.0)	13 (14.0)	33 (35.5)	60 (64.5)
Without siblings						
Yes	806 (77.1)	240 (22.9)	1029 (79.2)	270 (20.8)	487 (37.5)	812 (62.5)
No	951 (73.2)	348 (26.8)	834 (79.7)	212 (20.3)	391 (37.4)	655 (62.6)
Husband without siblings						
Yes	887 (75.6)	286 (24.4)	942 (80.4)	230 (19.6)	422 (36.0)	750 (64.0)
No	870 (74.2)	302 (25.8)	921 (78.5)	252 (21.5)	456 (38.9)	717 (61.1)
Marital status						
Unmarried	30 (61.2)	19 (38.8)	39 (79.6)	10 (20.4)	25 (51.0)	24 (49.0)
Primary marriage	1659 (75.2)	546 (24.8)	1746 (79.2)	459 (20.8)	820 (37.2)	1385 (62.8)
Remarried	51 (72.9)	19 (27.1)	59 (84.3)	11 (15.7)	23 (32.9)	47 (67.1)
Divorced or Widowed	17 (81.0)	4 (19.0)	19 (90.5)	2 (9.5)	10 (47.6)	11 (52.4)
Education level						
Basic education	290 (72.1)	112 (27.9)	371 (92.3)	31 (7.7)	134 (33.3)	268 (66.7)
Secondary education	262 (74.0)	92 (26.0)	316 (89.3)	38 (10.7)	130 (36.7)	224 (63.3)
Higher education	1205 (75.8)	384 (24.2)	1176 (74.0)	413 (26.0)	614 (38.6)	975 (61.4)
Residence						
Urban	1414 (75.2)	466 (24.8)	410 (88.2)	55 (11.8)	170 (36.6)	295 (63.4)
Rural	343 (73.8)	122 (26.2)	1453 (77.3)	427 (22.7)	708 (37.7)	1172 (62.3)
The per capital monthly income of the family						
<¥4500	467 (76.4)	144 (23. 6)	503 (82.3)	108 (17.7)	234 (38.3)	377 (61.7)
¥4500 and ¥9000	759 (76.7)	230 (23.3)	809 (81.8)	180 (18.2)	373 (37.7)	616 (62.3)
>¥9000	531 (71.3)	214 (28.7)	551 (74.0)	194 (26.0)	271 (36.4)	474 (63.6)
Occupation						
Rural migrant workers	91 (77.1)	27 (22.88)	115 (97.5)	3 (2.5)	53 (44.9)	65 (55.1)
Urban and rural unemployed	388 (70.2)	165 (29.8)	491 (88.8)	62 (11.2)	204 (36.9)	349 (63.1)
Industrial workers of Non-agricultural registered permanent residence	39 (78.0)	11 (22.0)	35 (70.0)	15 (30.0)	20 (40.0)	30 (60.0)
Individual business	146 (73.4)	53 (26.6)	179 (89.9)	20 (10.1)	64 (32.2)	135 (67.8)
Business services staff	119 (76.8)	36 (23.2)	125 (80.7)	30 (19.3)	60 (38.7)	95 (61.3)
Civil servants	324 (81.4)	74 (18.6)	293 (73.6)	105 (26.4)	153 (38.4)	245 (61.6)
Senior managers and Middle-level managers in large and medium enterprises	78 (81.3)	18 (18.7)	64 (66.7)	32 (33.3)	39 (40.6)	57 (59.4)
Private entrepreneur	59 (67.8)	28 (32.2)	73 (83.9)	14 (16.1)	21 (24.1)	66 (75.9)
Professionals	186 (76.2)	58 (23.8)	175 (71.7)	69 (28.3)	86 (35.3)	158 (64.7)
Clerk	106 (76.3)	33 (23.7)	78 (56.1)	61 (43.9)	53 (38.1)	86 (61.9)
Students	15 (100.0)	0 (0.0)	9 (60.0)	6 (40.0)	4 (26.7)	11 (73.3)
Other	206 (70.8)	85 (29.2)	226 (77.7)	65 (22.3)	121 (41.6)	170 (58.4)
Occupational category						
Non-manual	914 (77.6)	264 (22.4)	871 (73.9)	307 (26.1)	420 (35.7)	758 (64.4)
Manual	249 (77.1)	74 (22.9)	275 (85.1)	48 (14.9)	133 (41.2)	190 (58.8)
Unemployed	388 (70.2)	165 (29.8)	491 (88.8)	62 (11.2)	204 (36.9)	349 (63.1)
Others	206 (70.8)	85 (29.2)	226 (77.7)	65 (22.3)	121 (41.6)	170 (58.4)
Trimester of pregnancy						
First trimester	216 (73.7)	77 (26.3)	232 (79.2)	61 (20.8)	119 (40.6)	174 (59.4)
Second trimester	540 (77.0)	161 (23.0)	564 (80.5)	137 (19.5)	274 (39.1)	427 (60.9)
Third trimester	1001 (74.1)	350 (25.9)	1067 (79.0)	284 (21.0)	485 (35.9)	866 (64.1)

**Table 4:** Generalized linear model for identifying factors that affect TV viewing times, computer viewing times, mobile viewing times, and total viewing time among pregnant women, China, 2015.

Parameter	TV Viewing Times		Computer Usage Times		Mobile Phone Usage Times		The Total Time	
	Estimate (*SE*)	*p*	Estimate (*SE*)	*p*	Estimate (*SE*)	*p*	Estimate (*SE*)	*p*
TV viewing times	-	-	−0.059 (0.028)	0.035	0.134 (0.027)	<0.001	-	-
Computer viewing times	−0.032 (0.015)	0.035	-	-	0.049 (0.020)	0.015	-	-
Mobile phone usage times	0.080 (0.016)	<0.001	0.053 (0.022)	0.015	-	-	-	-
Pregnant women in second pregnancy vs. Pregnant women in first pregnancy	−0.288 (0.087)	0.0009	−0.461 (0.117)	<0.001	0.230 (0.113)	0.041	−0.523 (0.190)	0.006
Level 3A hospital vs. Level 2B hospitals and below	0.118 (0.123)	0.339	−0.027 (0.166)	0.869	−0.066 (0.160)	0.681	0.026 (0.272)	0.924
Level 2A hospitals vs. Level 2B hospitals and below	−0.187 (0.152)	0.220	−0.577 (0.205)	0.005	0.155 (0.197)	0.431	−0.616 (0.335)	0.066
Manual vs. Non-manual	0.103 (0.113)	0.360	−0.312 (0.152)	0.040	−0.502 (0.146)	0.001	−0.778 (0.248)	0.002
Unemployed vs. Non-manual	0.270 (0.095)	0.005	−0.722 (0.128)	<0.001	−0.312 (0.124)	0.012	−0.798 (0.208)	0.0001
Others vs. Non-manual	0.229 (0.110)	0.038	−0.257 (0.149)	0.084	−0.230 (0.143)	0.109	−0.274 (0.243)	0.259
Non-Han nationality vs. Han nationality	−0.161 (0.178)	0.366	−0.175 (0.240)	0.467	0.191 (0.231)	0.409	−0.137 (0.393)	0.728
Without siblings vs. With siblings	−0.102 (0.072)	0.157	−0.105 (0.097)	0.282	0.059 (0.094)	0.529	−0.151 (0.159)	0.342
Husband was not single child vs. Husband was single child	−0.041 (0.071)	0.565	−0.076 (0.096)	0.431	0.013 (0.092)	0.889	−0.107 (0.157)	0.495
Primary marriage vs. Divorced or Widowed	0.585 (0.368)	0.112	0.560 (0.496)	0.259	0.831 (0.477)	0.082	2.160 (0.810)	0.008
Unmarried vs. Divorced or Widowed	0.692 (0.436)	0.113	0.791 (0.588)	0.179	0.138 (0.566)	0.808	1.720 (0.961)	0.074
Remarried vs. Divorced or Widowed	0.446 (0.416)	0.285	0.473 (0.564)	0.402	1.060 (0.542)	0.051	2.180 (0.920)	0.018
Secondary education vs. Basic education	−0.117 (0.124)	0.343	0.094 (0.167)	0.573	−0.024 (0.161)	0.880	−0.060 (0.273)	0.827
Higher education vs. Basic education	−0.296 (0.111)	0.008	0.553 (0.150)	0.0002	−0.287 (0.144)	0.047	−0.087 (0.244)	0.723
Urban vs. Rural	−0.077 (0.095)	0.421	0.082 (0.129)	0.523	0.002 (0.124)	0.986	0.002 (0.210)	0.991
¥4500 and ¥9000 vs. <¥4500	0.046 (0.090)	0.604	−0.215 (0.121)	0.075	−0.100 (0.116)	0.388	−0.283 (0.197)	0.151
>¥9000 vs. <¥4500	0.232 (0.099)	0.020	0.014 (0.135)	0.919	−0.089 (0.129)	0.490	0.166 (0.220)	0.450
Second trimester vs. First trimester	0.033 (0.116)	0.779	0.024 (0.157)	0.877	−0.083 (0.151)	0.585	−0.034 (0.257)	0.896
Third trimester vs. First trimester	0.097 (0.108)	0.368	−0.004 (0.146)	0.977	0.088 (0.140)	0.528	0.203 (0.238)	0.394
26–35 years old vs. 18–25 years old	0.225 (0.084)	0.007	0.293 (0.114)	0.010	−0.217 (0.109)	0.047	0.298 (0.185)	0.109
36–45 years old vs. 18–25 years old	0.395 (0.177)	0.026	0.270 (0.239)	0.259	−0.275 (0.230)	0.233	0.394 (0.391)	0.314

**Table 5:** Multivariate logistic regression model for prolonged TV, computer, and mobile viewing times among pregnant women, China, 2015.

TV Viewing Times		Computer Usage Times		Mobile Phone Usage Times	
Parameter	*OR*^a1^ (95% *CI*)	Parameter	*OR*^a2^ (95% *CI*)	Parameter	*OR*^a3^ (95% *CI*)
Mobile phone usage time		Mobile phone usage time		TV viewing time	
≤1 h	1.00	≤1 h	1.00	≤2 h	1.00
>1 h	1.52 (1.25–1.86)	>1 h	1.32 (1.06–1.64)	>2 h	1.56 (1.27–1.91)
Hospital level		Hospital level		Computer viewing time	
Level 2B hospitals and below	1.00	Level 2B hospitals and below	1.00	≤2 h	1.00
Level 3A hospital	1.11 (0.80–1.56)	Level 3A hospital	0.88 (0.61–1.27)	>2 h	1.32 (1.06–1.65)
Level 2 A hospitals	0.76 (0.50–1.17)	Level 2 A hospitals	0.53 (0.31–0.89)	-	-
Occupational category		Occupational category		Occupational category	
Non-manual	1.00	Non-manual	1.00	Non-manual	1.00
Manual	1.17 (0.86–1.58)	Manual	0.74 (0.52–1.06)	Manual	0.72 (0.55–0.94)
Unemployed	1.65 (1.30–2.10)	Unemployed	0.51 (0.37–0.70)	Unemployed	0.84 (0.67–1.05)
Others	1.42 (1.06–1.89)	Others	0.86 (0.62–1.18)	Others	0.74 (0.56–0.96)
Without siblings		Without siblings		Without siblings	-
No	1.00	No	1.00	No	-
Yes	0.79 (0.65–0.95)	Yes	0.76 (0.61–0.94)	Yes	-
The per capital monthly income of the family		The per capital monthly income of the family		The per capital monthly income of the family	-
<4500¥	1.00	<4500¥	1.00	<4500¥	-
4500¥ and 9000 ¥	1.03 (0.81–1.32)	4500¥ and 9000 ¥	0.69 (0.52–0.92)	4500¥ and 9000 ¥	-
>9000¥	1.42 (1.09–1.86)	>9000¥	0.92 (0.68–1.23)	>9000¥	-
Parity	-	Parity		Parity	
Pregnant women in first pregnancy	-	Pregnant women in first pregnancy	1.00	Pregnant women in first pregnancy	1.00
Pregnant women in second pregnancy	-	Pregnant women in second pregnancy	0.56 (0.42–0.74)	Pregnant women in second pregnancy	1.25 (1.01–1.55)
Education level	-	Education level		Education level	
Basic education	-	Basic education	1.00	Basic education	1.00
Secondary education	-	Secondary education	1.34 (0.81–2.23)	Secondary education	0.83 (0.61–1.13)
Higher education	-	Higher education	2.72 (1.78–4.16)	Higher education	0.73 (0.56–0.95)
Age	-	Age		Age	
18–25 years old	-	18–25 years old	1.00	18–25 years old	1.00
26–35 years old	-	26–35 years old	1.33 (1.02–1.74)	26–35 years old	0.80 (0.65–0.98)
36–45 years old	-	36–45 years old	1.09 (0.60–1.95)	36–45 years old	0.70 (0.45–1.08)
Marital status	-	Marital status	-	Marital status	
Primary marriage	-	Primary marriage	-	Primary marriage	1.00
Unmarried	-	Unmarried	-	Unmarried	0.48 (0.27–0.85)
Remarried	-	Remarried	-	Remarried	1.14 (0.67–1.92)
Divorced or Widowed	-	Divorced or Widowed	-	Divorced or Widowed	0.66 (0.28–1.58)
Residence	-	Residence	-	Residence	-
Rural	-	Rural	1.00	Rural	-
Urban	-	Urban	1.34 (0.97–1.87)	Urban	-

^a1^ Adjusted for mobile phone usage times, hospital level, occupational category, without siblings, and the per capita monthly income of the family; ^a2^ Adjusted for mobile phone usage times, age, hospital level, occupational category, without siblings, education level, residence, the per capita monthly income of the family, and trimester of pregnancy; ^a3^ Adjusted for TV viewing times, computer usage times, age, occupational category, marital status, education level, and trimester of pregnancy.
